# Decentralization matters – Differently organized mental health services relationship to staff competence and treatment practice: the VELO study

**DOI:** 10.1186/1752-4458-3-9

**Published:** 2009-05-18

**Authors:** Svein Bjorbekkmo, Lars H Myklebust, Reidun Olstad, Stian Molvik, Asle Nymann, Knut Sørgaard

**Affiliations:** 1 Nordland Hospital Trust Vesteraalen, DPS, N-8450 Stokmarknes, Norway; 2 Psychiatric Research Center of North Norway, University Hospital of North Norway, Postboks 6124, N-9291 Tromsø, Norway; 3 University of Tromsø, Institute of Clinical Medicine, Dept. of Clinical Psychiatry, N-9291 Tromsø, Norway; 4 Nordland Hospital Trust Bodø, Psychiatric Division, Kløveraasveien 1, N-8092 Bodø, Norway; 5 Nordland Hospital Trust Lofoten, DPS, N-8372 Gravdal, Norway; 6 Psychiatric Research Center of North Norway, Nordland Hospital Trust, Kløveraasveien 1, N-8092 Bodø, Norway

## Abstract

**Background:**

The VELO study is a comparative study of two Community Mental Health Centres (CMHC) in Northern Norway. The CMHCs are organized differently: one has no local inpatient unit, the other has three. Both CMHCs use the Central Mental Hospital situated rather far away for compulsory and other admissions, but one uses mainly local beds while the other uses only central hospital beds. In this part of the study the ward staffs level of competence and treatment philosophy in the CMHCs bed units are compared to Central Mental Hospital units. Differences may influence health service given, resulting in different treatment for similar patients from the two CMHCs.

**Methods:**

167 ward staff at Vesterålen CMHCs bed units and the Nordland Central Mental Hospital bed units answered two questionnaires on clinical practice: one with questions about education, work experience and clinical orientation; the other with questions about the philosophy and practice at the unit. An extended version of Community Program Philosophy Scale (CPPS) was used. Data were analyzed with descriptive statistics, non-parametric test and logistic regression.

**Results:**

We found significant differences in several aspects of competence and treatment philosophy between local bed units and central bed units. CMHC staff are younger, have shorter work experience and a more generalised postgraduate education. CMHC emphasises family therapy and cooperation with GP, while Hospital staff emphasise diagnostic assessment, medication, long term treatment and handling aggression.

**Conclusion:**

The implications of the differences found, and the possibility that these differences influence the treatment mode for patients with similar psychiatric problems from the two catchment areas, are discussed.

## Background

During the last decades, the developmental trend in psychiatric services in western countries has been away from large psychiatric hospitals to more locally based psychiatric services [1]. The community based services have become the cornerstone of the mental health treatment systems, and the role of the central hospitals has changed towards delivering more time limited back-up service in periods of crises for the patients and services for patients with special needs [2].

This development may be seen partly as a consequence of disillusionment concerning large psychiatric hospitals' suitability for modern treatment of psychiatric illnesses, and partly as related to a general socio-political trend favouring more locally based solutions [3]. Central in this development is the idea that it is better for the patient to stay in and receive treatment in their community, and that this will ease access to the services, lead to better cooperation between service levels and reduce stigma.

This developmental trend has also characterised the development of psychiatric services in Norway during the last three decades [4]. The number of patients in central psychiatric institutions has been reduced, and specialized outpatient and inpatient services are transferred to Community Mental Health Centres (CMHC), often integrated in local general hospitals. In addition, community based mental health services provided by the municipalities have been strengthened. The role of the CMHC is described by the Norwegian Directorate of Health and Social Welfare [5,6] as a specialist health service collaborating with the municipal organisations and backed by specialized hospital services. Initially there were no strong directives from the Norwegian health authorities as to how the decentralized specialist services (CMHC) should be organized. This led to a variety of organizational models, often expressing the treatment philosophy of local professionals.

Mental health care is almost exclusively based on human resources (human technology), and seldom, as may be the case in other areas of health care, on instrumental technology. This is the case for diagnostic processes as well as for therapy. It is a central challenge in the mental health field to make sure there is staff with optimal education and experience, and that the organizational climate and the motivation of the personnel facilitates the conditions for the best outcome of the treatment.

Human resources are not static, but will deteriorate if there is not a continual process of maintaining and refreshing professional knowledge and skills. According to Thornicroft and Tansella [7], there is a striking lack of research in this field. Rosenvinge [8] describes three central elements of clinical competence. First knowledge, that is basic professional education and relevant postgraduate education. Second are clinical skills, developed in a professional setting through encounter with patients and from supervision, and third, there are professional ethics in the sense of a reflective relation to knowledge and to one's own professional behaviour. Roth and Fonagy [9] conclude in a study of psychotherapy that good outcome of psychotherapy is more strongly related to competence in terms of skills and knowledge than to length of experience.

To describe and characterize psychiatric services has been called "a difficult and dangerous task" [10]. It may be "difficult" because we do not have good enough theoretical concepts and methods, and "dangerous" because it describes treatment practice instead of just counting beds or consultations. It has therefore been necessary to develop instruments and methods that can be used to describe and differentiate between mental health practices and programs in a valid, reliable and time saving/economical manner [11-13]. Such instruments are necessary tools for service planners and clinical leaders in their efforts to evaluate and develop the services. Different scales for description of organizational culture, climate and treatment philosophy at different mental health services have been developed [14-19]. Hargreaves and Jerrell [20] at the University of California introduced in 1991 the Community Program Philosophy Scale (CPPS), later renamed Community Program Practice Scale [21]. The scale intends to be an instrument for the staff to describe treatment philosophy and treatment practice at their psychiatric outpatient service. The scale has been translated to Norwegian by Ruud [22] and augmented with 40 items adapted to inpatient staff.

Variations in the organizational structure and treatment philosophy of Norwegian CMHCs are not uncommon. In Northern Norway, two of the CMHC units developed along very different lines with regards to organization, staffing, clinical services, use of and access to inpatient treatment [23]. In these two organizations, although serving close to equal sized populations in demographically similar areas, the staffing ratio is about 3:1. The ratio for use of psychiatric beds are equal in the two areas, about 1 bed pr 1000 inhabitants [24]. But whereas one of the centres has no psychiatric inpatient services and uses beds in the county's central psychiatric hospital, the other has established 3 small decentralized inpatient units in their catchment area and uses beds at the Central Mental Hospital to a very limited extent and only for very selected purposes such as compulsory treatment and severe eating disorders. Thus, in one of the centres the inpatient services are integrated in a comprehensive community mental health centre that works in accordance with community mental health principles, whereas the other uses the inpatient services in a traditional and far-off mental hospital.

In this article, we will focus on the relationship between differently organized treatment systems and the philosophy of the treatment staff as measured by CPPS. Two groups of inpatient staff are studied: (i) those employed at the CMHC with integrated and decentralised inpatient units, and (ii) the staff at the wards of the county Central Mental Hospital (Nordland Hospital) that primarily serves the CMHC units that operate without access to local inpatient facilities. To our knowledge no similar comparisons have been done before, and the study has an explorative character. We expected to find some systematic differences in program philosophy and practice between the two groups of inpatient staff, reflecting the different context of their clinical work. We also wanted to examine differences in the level of experience and competence between the ward staff in local and central inpatient units.

## Methods

### The context of the study

Vesterålen CMHS has a catchment area of 30500 inhabitants, and consists of an outpatient clinic and an inpatient ward consisting of three small units with 8, 6 and 6 beds. The three inpatient units are decentralized in three different municipalities. The inpatient units are multi-purpose, open, short stay psychiatric units, mainly used for shorter crises and relief stays, but occasionally for longer stays for several months. The inpatient staff work together with outpatient staff in the patients' home environment, and doctors and psychologists at the outpatient clinic also have the responsibility for treatment in the inpatient units. The full-time equivalent staff is 77.

All compulsory and some acute hospitalizations from the region are remitted to the psychiatric hospital in Bodø. Some patients are also referred to the Central Mental Hospital for long term treatment and for rehabilitation. The distance to Bodø from the region is about 1/2 – 1 hour by plane or 4–5 hours by car/ambulance.

Three wards at the Central Mental Hospital participated in the study: (i) An acute ward with 10 beds, 33 staff including 6 psychologists and psychiatrists. The ward is used mainly for short term stay for acutely ill patients. (ii) A general inpatient ward with 24 beds, 52 staff including 13 psychologists and psychiatrists. The ward treats patients with mood disorders, post-traumatic disorders, eating disorders and personality problems. Stays are usually for three months, occasionally longer. (iii) A rehabilitation ward with 37 beds. The number of staff is 117 including 11 psychologists and psychiatrists. The ward serves patients with serious mental disorders (mainly psychosis) on a long term (up to one year) basis.

In this article the term CMHC-units refers to the decentralised units in Vesterålen, whereas hospital wards refers to the Central Mental Hospital's acute, general and rehabilitation units in Bodø. (see Table 1)

**Table 1:** Organizational outline of Nordland Hospital, psychiatric division.

		**Vesterålen CMHC**	**Nordland Hospital acute**	**Nordland Hospital general**	**Nordland Hospital rehab**.
Units	Outpatient clinic	1	-	-	-
	Daycare centres	-	-	-	-
	Bed units	3	1	2	7
	Number of beds	20	10	24	37
					
Staff at inpatient units	Number of ward staff	57	27	39	106
	Ward personnel number per bed	2,85	2,75	1,62	2,95*
	Treatment staff rate (doctor/psychologist a.o) per bed	0,2	0,6	0,5	0,3
Total equivalent full-time staff		77	33	52	117

*ranges from 1,3 to 4,6 (4,6 in a security ward)

### Design

A cross-sectional design was used. Leaders, clinicians and ward staff at the Vesterålen CMHS, and the acute, general and rehabilitation units at the psychiatric division at Nordland Hospital in Bodø, were informed about the study and asked to participate. It was emphasised that participation was voluntary and that all data were confidential. After accepting and signing an agreement form, they were delivered the two forms. 252 employees answered the questionnaires. To examine our main hypothesis, that there is a connection between organisational context and treatment philosophy, we chose the ward staff at the inpatient units in Vesterålen CMHC and the three units at the psychiatric department of Nordland Hospital in Bodø. 167 ward staff answered the questionnaires. The response rate was 94% in CMHC and 71% in Hospital units.

### Instruments

To study competence a modified version of a questionnaire ("Assessment of competence in the health services") developed by Ruud [22] was used. This form includes questions about age, sex, basic and postgraduate education, amount of work experiences, competence, clinical orientation and task priorities at the work place.

The Community Program Philosophy Scale (CPPS) measures practice and treatment philosophy at the working place. An extended Norwegian version of CPPS was used. The first 80 items in 20 sub-scales are identical with Jerrells and Hargreaves original scale [20]. In addition 40 items in 10 sub-scales developed by Ruud [22] are included. These items are specially designed for inpatient units, and have sub-scales for sheltering, handling aggression, amount of care, etc. Ward staff were asked to describe climate, philosophy and practice at their unit by responding to the 120 statements on a five point Likert scale (1 = strongly disagree, 5 = strongly agree). Statements are grouped into 30 sub-scales with 4 statements in each. Five of the sub-scales are about internal climate while the rest of the sub-scales describe treatment practice. We chose to use the extended Norwegian version (NeCPPS) because it has scales for inpatient practice, and offers an opportunity to compare our results to international studies as well as research from Norwegian mental health services. The psychometric qualities of the Norwegian version are equal to the American version as measured by Cronbach's alpha [22].

The Regional Ethics Committee and The Norwegian Social Science Data Services approved the study.

### Data analysis

Statistical computations were performed with SPSS statistical package version 16.0.

Descriptive statistics were used to describe the sample in terms of personal characteristics, education and work experience. Parametric (t-test), non-parametric tests (Mann-Whitney U, Chi-square) and logistic regression were used for studying between-group differences.

## Results

### Competence and Experience

Table 2 shows data from the competence questionnaire and socio-demographic data for ward staff at Vesterålen CMHC and the three psychiatric units at Nordland Hospital.

**Table 2:** Ward staff competence and experience at CMHC and hospital bed units

		**Vesterålen CMHC**	**Nordland Hospital acute**	**Nordland Hospital general**	**Nordland Hospital rehab**
	Response rate (%)	94	80	64	72
					
Sex (%)	Women	83,3	65,0	95,2	*61,1
					
Age (%)	< 30	13,0	5,0	0	4,2
	30–39	24,1	30,0	28,6	26,4
	40–49	40,7	30,0	28,6	31,9
	50–59	20,4	35,0	33,3	27,8
	> 60	1,9	0	9,5	4,2
					
Education (%)	Nurses	29,6	60,0	71,4	41,7
	Social educator	9,3	0	0	0
	Social workers	3,7	10,0	4,8	11,1
	Teachers	9,2	0	0	8,3
	Other university college education	3,7	0	0	9,0
	*Total university college education:*	*55,5*	**70,0	**76,2	**70,1
	Assistant nurses	18,5	15,0	4,8	19,4
	Others	18,5	10,0	9,5	5,6
	Assistents	5,6	5,0	4,8	2,8
	*Total without univ. college education:*	*42,6*	*30*	*19,1*	*27,8*
					
Postgraduate education (%)	Specialist within own profession	42,0	55,0	65,0	41,0
	Supervisory competence within own profession	1,9	5,0	9,5	9,7
	Competence in cognitive therapy	11,0	20,0	23,8	27,9
	Psychosocial rehabilitation of psychoses competence program	18,5	0	4,6	8,0
					
Work experience	Total (mean years)	19,6	20,3	23,2	20,9
	Total (range)	(1–40)	(2–42)	(9–36)	(2–46)
	In psychiatric care (mean years)	6,1	*10,1	**14,3	**13,7
	At present work place (mean years)	5,3	**10,2	8,6	**10,6

* significantly different from Vesterålen CMHC p < 0,05

** significantly different from Vesterålen CMHC p < 0,005

Comparing the ward staff in the CMHC to the ward staff at the three hospital units, we find that the percentage of women is higher at the CMHC wards and the Hospital general ward compared to Hospital acute and rehabilitation wards. The staff at the CMHC is also younger. Mean years of practice in the mental health field is higher at all hospital units. Here we also find the highest proportion of registered nurses among the staff. The proportion of staff with postgraduate education is generally high, and there are many specialists within own profession. Competence in cognitive psychology is more common at the hospital units, while the CMHC staff have more generalized postgraduate education aimed at treating and caring for persons with severe mental illness. The number per bed of doctors/psychiatrists and psychologist is also higher at the hospital units.

### Therapeutic Profile

CPPS ratings show that all units rate help with socio-economic rights, out of office orientation, teamwork, user participation and medication emphasis high, as well as the climate scales involvement and staff support high or very high. Psychotherapy is generally rated in the low to medium range.

Univariate analyses (T-test/Mann-Whitney) were performed on all the the CPPS sub-scales. A number of significant differences between the CMHC wards and the Central Mental Hospital wards were found as shown in table 3 and 4.

**Table 3:** CPPS subcale scores; means and standard deviations. Significant differences in ward staff ratings between CMHC inpatient units and hospital units.

**Variable**	**Vesterålen CMHC**	**Nordland Hospital acute**	**Nordland Hospital general**	**Nordland Hospital rehab**	**Total**
**Climate scales**					
1. Innovation	3,4 (0,5)	3,0 (0,4)*	3,5 (0,5)	3,3 (0,6)	3,3 (0,6)
2. Involvement	4,4 (0,5)	4,3 (0,4)	4,5 (0,4)	4,1 (0,5)*	4,3 (0,5)
3. Clarity	3,3 (0,7)	3,5 (0,5)	3,8 (0,4)*	3,7 (0,6)**	3,6 (0,6)
4. Mutual staff support	4,1 (0,5)	4,2 (0,4)	4,2 (0,4)	4,1 (0,5)	4,1 (0,5)
5. Supervisory support	3,7 (0,5)	3,7 (0,7)	4,0 (0,5)	3,6 (0,6)	3,7 (0,5)
**Practice scales**					
6. Out-of office orientation	4,0 (0,5)	3,9 (0,6)	3,8 (0,5)	4,0 (0,6)	4,0 (0,6)
7. Team vs individual	4,0 (0,6)	4,2 (0,4)*	4,2 (0,4)	4,2 (0,6)*	4,1 (0,5)
8. Help with housing	3,6 (0,6)	3,6 (0,6)	2,6 (0,6)**	4,1 (0,6)**	3,7 (0,8)
9. Severe mental disorders	3,9 (0,5)	4,0 (0,5)	3,5 (0,4)**	3,9 (0,5)	3,9 (0,5)
10. Involve family	3,5 (0,7)	3,0 (0,8)*	3,4 (0,4)	3,5 (0,8)	3,4 (0,7)
11. Substance abuse emphasis	3,6 (0,6)	3,9 (0,5)	3,4 (0,5)	3,8 (0,8)	3,7 (0,7)
12 Help with socio-econom rights	4,2 (0,5)	4,3 (0,4)	4,3 (0,4)	4,5 (0,4)*	4,3 (0,4)
13 Emergency accessability	3,3 (0,7)	3,4 (0,5)	3,5 (0,6)	3,4 (0,7)	3,4 (0,7)
14 Follow up emphasis	3,6 (0,6)	3,6 (0,5)	3,3 (0,6)*	4,2 (0,6)**	3,8 (0,6)
15 Cooperation with externals	3,6 (0,5)	3,3 (0,4)	3,4 (0,6)	3,6 (0,6)	3,5 (0,6)
16 Users perspective	3,9 (0,5)	3,7 (0,5)	4,1 (0,5)	3,9 (0,6)	3,9 (0,6)
17 Work rehabilitation	3,5 (0,4)	3,0 (0,6)**	3,2 (0,6)	3,8 (0,5)**	3,5 (0,6)
18 Psychoterapy emphasis	2,8 (0,5)	3,0 (0,5)	3,0 (0,4)	2,5 (0,7)**	2,7 (0,6)
19 Medication emphasis	3,6 (0,5)	4,1 (0,3)**	3,6 (0,5)	4,0 (0,7)**	3,8 (0,6)
20. Long term emphasis	2,9 (0,6)	2,2 (0,4)**	2,3 (0,7)**	4,0 (0,5)**	3,2 (0,9)
21. Handle aggression	2,9 (0,6)	4,3 (0,4)**	2,4 (0,7)*	3,5 (1,0)**	3,2 (0,9)
22 Evaluation, diagnostic empha	3,3 (0,7)	2,8 (0,7)*	4,1 (0,6)**	3,7 (0,6)**	3,5 (0,7)
23. Support emphasis	3,6 (0,5)	4,1 (0,5)**	3,2 (0,6)**	3,9 (0,6)*	3,8 (0,6)
24. Sheltering	3,5 (0,5)	4,3 (0,4)**	2,5 (0,6)**	3,4 (0,8)	3,4 (0,8)
25. Contact GP	3,5 (0,6)	3,1 (0,5)*	3,4 (0,4)	3,2 (0,7)*	3,3 (0,6)
26. Responsibility for untreated	2,8 (0,5)	3,3 (0,6)**	2,4 (0,5)**	2,3 (0,5)**	2,6 (0,6)
27. Working through emphasis	3,3 (0,5)	2,9 (0,6)**	3,3 (0,6)	3,6 (0,6)**	3,4 (0,6)
28. Family therapy emphasis	3,5 (0,6)	2,6 (0,6)**	3,3 (0,4)	3,2 (0,7)*	3,2 (0,7)
29. Group therapy emphasis	3,6 (0,6)	3,1 (0,6)**	4,4 (0,6)**	3,4 (0,8)	3,5 (0,8)
30. Skill training emphasis	3,3 (0,5)	2,8 (0,4)**	3,6 (0,6)	3,6 (0,6)*	3,4 (0,6)

* significantly different from Vesterålen CMHC p < 0,05 Mann Whitney U Test

** significantly different from Vesterålen CMHC p < 0,005 Mann Whitney U Test

Range emphasis: very small 1 – 1,8, small 1,9 – 2,6, moderate 2,7 – 3,4, large 3,5 – 4,2, very large 4,3 – 5,0

**Table 4:** Scores on CPPS subcales; Univariate analyses: means and standard deviation.

**Variable**	**Vesterålen CMHC**	**Nordland Hospital units**	**Total**	**Asymp sig. 2 tailed Mann-Whitney**
**Climate scales**				
1. Innovation	3,4 (0,5)	3,3 (0,6)	3,3 (0,6)	.196
2. Involvement	4,4 (0,5)	4,2 (0,5)	4,3 (0,5)	.053
3. Clarity	3,3 (0,7)	3,7 (0,6)*	3,6 (0,6)	.002**
4. Mutual staff support	4,1 (0,5)	4,1 (0,5)	4,1 (0,5)	.797
5. Supervisory support	3,7 (0,5)	3,6 (0,6)	3,7 (0,5)	.578
**Practice scales**				
6. Out-of office orientation	4,0 (0,5)	3,9 (0,6)	4,0 (0,6)	.350
7. Team vs individual	4,0 (0,6)	4,2 (0,5)**	4,1 (0,5)	.003**
8. Help with housing	3,6 (0,6)	3,7 (0,8)	3,7 (0,8)	.180
9. Severe mental disorders	3,9 (0,5)	3,8 (0,5)	3,9 (0,5)	.504
10. Involve family	3,5 (0,7)	3,4 (0,8)	3,4 (0,7)	.281
11. Substance abuse emphasis	3,6 (0,6)	3,7 (0,7)	3,7 (0,7)	.165
12 Help with socio-econom rights	4,2 (0,5)	4,4 (0,4)*	4,3 (0,4)	.046*
13 Emergency accessability	3,3 (0,7)	3,4 (0,7)	3,4 (0,7)	.630
14 Follow up emphasis	3,6 (0,6)	3,9 (0,6)*	3,8 (0,6)	.014*
15 Cooperation with externals	3,6 (0,5)	3,5 (0,6)	3,5 (0,6)	.696
16 Users perspective	3,9 (0,5)	3,9 (0,6)	3,9 (0,6)	.596
17 Work rehabilitation	3,5 (0,4)	3,5 (0,6)	3,5 (0,6)	.348
18 Psychoterapy emphasis	2,8 (0,5)	2,7 (0,7)	2,7 (0,6)	.171
19 Medication emphasis	3,6 (0,5)	4,0 (0,6)**	3,8 (0,6)	.000**
20. Long term emphasis	2,9 (0,6)	3,3 (1,0)**	3,2 (0,9)	.002**
21. Handle aggression	2,9 (0,6)	3,4 (1,0)**	3,2 (0,9)	.000**
22 Evaluation, diagnostic empha	3,3 (0,7)	3,6 (0,8)**	3,5 (0,7)	.002**
23. Support emphasis	3,6 (0,5)	3,8 (0,6)**	3,7 (0,6)	.039*
24. Sheltering	3,5 (0,5)	3,4 (0,9)	3,4 (0,8)	.559
25. Contact GP	3,5 (0,6)*	3,2 (0,6)	3,3 (0,6)	.029*
26. Responsibility for untreated	2,8 (0,5)**	2,5 (0,7)	2,6 (0,6)	.000**
27. Working through emphasis	3,3 (0,5)	3,4 (0,6)	3,4 (0,6)	.151
28. Family therapy emphasis	3,5 (0,6)**	3,1 (0,7)	3,2 (0,7)	.000**
29. Group therapy emphasis	3,6 (0,6)	3,5 (0,9)	3,5 (0,8)	.577
30. Skill training emphasis	3,3 (0,5)	3,4 (0,6)	3,4 (0,6)	.512

Significant differences in ward staff ratings between CMHC inpatient units and hospital units.

* significantly higher p < 0,05 Mann Whitney U Test

** significantly higher p < 0,005 Mann Whitney U Test

Range emphasis: very small 1 – 1,8, small 1,9 – 2,6, moderate 2,7 – 3,4, large 3,5 – 4,2, very large 4,3 – 5.0

On the ward level, the main differences were between the CMHC units and the hospital acute ward (significant differences on 15 of 30 sub-scales) and between the CMHC units and the hospital rehabilitation wards (18 of 30 sub-scales). The differences were most evident when the CMHCs were compared to the rehabilitation units. The most substantial differences were found on scales like emphasis on help with housing, work rehabilitation, medication, evaluation, working through, and above all on long term treatment. Hospital rehabilitation ward all ranked these higher than the CMHC. The hospital acute ward and rehabilitation units were similar in that they put significantly more importance on medication and handling of aggression than the CMHC and the hospital general unit. Most similarities were found between the CMHC and the hospital general ward (significant differences on 11 of 30 scales).

In a logistic regression analysis, scales with univariate p-values < .20 were included in the analyses as potential explanatory variables. These variables were innovation, substance abuse emphasis, medication, long term emphasis, handle aggression, diagnostic emphasis, contact with GP and family therapy. Then a backward stepwise analysis with CMHC staff and Central hospital staff as the dependent variables (table 5) was performed. The table shows that hospital staff conceptualized their work more in terms of long term treatment, handling aggression, emphasis on evaluation/diagnostics and emphasis on medication, whereas their CMHC-inpatient colleagues emphasised use of family therapy and, to a somewhat lesser extent, contact with GPs.

**Table 5:** Logistic regression with CMHC-inpatients staff vs Hospital staff as dependent variable (N = 161)

**Variable**	**B**	**S.E**.	**Exp (B)**	***95% C.I for EXP (B)***	
19. Medication emphasis	.785	.381	2.193	1.038	4.632
20. Long term emphasis	.577	.251	1.780	1.089	2.909
21. Handle aggression	.492	.242	1.636	1.018	2.629
22 Evaluation, diagnostic empha	1.209	.338	3.349	1.726	6.498
25. Contact GP	-.725	.385	.484	.228	1.030
28. Family therapy emphasis	-1.275	.419	.279	.123	.634

## Discussion

In this study of competence and treatment philosophy or treatment profile we have compared the staff at three inpatient wards in a community mental health centre to the staff at the inpatient wards in a central mental hospital with regard to (a) demography, education, experience with work in mental health services and (b) on their ranking of the wards' treatment orientation. On competence the main differences were that the ward staff at the hospital generally has longer experience from working in psychiatric care, have longer and more medically oriented education and more specialized post graduate education.

The univariate analyses showed that on treatment philosophy, all units strongly emphasised help with socio-economic rights, out of office orientation, teamwork, user participation and medication. Psychotherapy was generally given a low priority.

One may wonder why, with the exception of medication, treatment approaches were not prioritized. All these treatment units are expected to give highly specialised services, but there seemed to be an informal agreement that organisational and practical approaches were more important than psychotherapy, group therapy and family therapy. It is noteworthy that psychotherapy, next to responsibility for the untreated, was given the lowest average rating of all the CPPS items. Neither was assessment (diagnosis, evaluation) considered especially important. The explanation for this may be that the participants in the study were ward staff, but it is a paradox that specialised treatment services seem to downplay the role of specific therapeutic approaches. The proportion of hospital ward staff with training in cognitive therapy was sizeable. A related question is the almost uniform importance attributed to medication in treatment units covering a rather heterogeneous patient population.

On the ward level we found, as might be expected, the largest differences in treatment philosophy between the hospital acute unit and the CMHC. Even though CMCH wards admit patients for shorter crisis stay, in contrast to hospital acute ward, they do not use coercive measures and do not receive patients for compulsory detention and treatment. On the other hand we would expect the hospital general unit and rehabilitation unit to fill functions more similar to the local CMHC units, because these units probably receive similar patient groups, patients not in need for compulsory detention, but in need for inpatient treatment. In one system they are hospitalized locally, in the other in central hospital. As expected, we found that the treatment philosophy surrounding the beds in Vesterålen was comparable to the treatment principles in the hospital general psychiatric unit. But the hospital rehabilitation units were more similar to the acute unit, putting significantly more weight on medication, on handling aggression and on support than the CMHC and the Hospital general unit. The general impression was that the CMHC staff were most similar to the hospital general unit staff both with regard to competence and treatment orientation. We also expected many similarities in competence and treatment philosophy between the CMHC wards and the hospital rehabilitation ward, because we thought patients in need for rehabilitation programs would be a relatively homogenious group. These expectations were not confirmed. The largest differences were found between the CMHC wards and hospital rehabilitation unit. Significant differences were found on treatment orientation scales like psychotherapy, medication and family therapy, and on cooperation with other parts of the health service (follow up, contact with GP), on threshold for intake, and above all, on long term treatment.

When we compare, the CPPS reference values from USA [21] show more emphasis on basic care, social support and medication in the USA values. This difference may be connected to dissimilarities between the Norwegian public health service and the American health service, and in differences in social legislation. In Norway, the most important single factor behind the deinstitutionalisation was the disability pension which, in addition to other social welfare laws, made living outside mental hospital possible for seriously mentally ill persons [3]. Higher population density in USA makes it more natural to develop more specialized programs for different groups of patients. In more scattered populated areas in Norway, it is necessary to give a public general psychiatric service. Alternative private health services, especially for the less severe psychiatric problems, are supposed to be better available in USA, while it is almost non-existent in the regions in Norway that we study [4].

When CMHC and hospital staff were used as a dependent variable in the logistic regression analysis, it became apparent that the main differences between the two groups were that the hospital staff conceptualized their work more in terms of long term treatment, handling aggression, emphasis on evaluation/diagnostics and emphasis on medication, whereas their CMHC-inpatient colleagues emphasised use of family therapy and, to a somewhat lesser extent, contact with GPs. In spite of many univariate similarities, there seemed to be a distinct hospital professional profile characterized by a more diagnostically and medically oriented approach directed at patients with aggression and long term problems. The importance of aggression may be caused by some of these wards often receiving patients who are dangerous to others or to themselves, more often acutely ill and disorganized. But many hospital inpatients do not suffer from such problems, and only one of the hospital wards in the present study accept acutely ill patients. The hospital profile becomes more evident when we compare it with the attitudes of the CMHC staff and their orientation towards family treatment and collaboration with GPs. In spite of considerable overlap in professional attitudes and philosophy the distinctness of the different profiles seems to be real, even though the mechanism behind them is not clear.

It has been argued that a decentralised mental health organization might have problems with recruitment of personnel with relevant competence and maintenance of a high professional standard [7,25]. To some extent we find support for this view. This might to some degree be balanced with an advantage in geographical closeness to municipality services and the patient's family and social network, giving better conditions for continuity of care than a more far away central hospital system might give. The differences in emphasis on family therapy, contact with GP, medication and long term treatment might reflect that the CMHC wards have a more resource and social network oriented practice looking for solutions in cooperation with the patient's local support system, while the hospital system focuses more on reduction of symptoms and strengthening of the patient's function through therapeutic efforts within the ward. These differences in treatment orientation might also reflect a conscious strategy to recruit professionals with different educational background and experience to CMHC-team than what is usual in traditional mental hospital teams. There is also a difference in postgraduate education between the CMHC and hospital units. CMHC emphasises generalized rehabilitation programs while hospital emphasises cogntive therapy. This may reflect an intended strategy or a difference in treatment orientation that is related to organizational frames or differences in professional background.

If reported treatment philosophy in our study influences and reflects actual treatment practice, this means that patients referred to the Central Mental Hospital units to some extent receive different treatment compared to the CMHC treated patients. We have mentioned that the acute ward at the hospital is used for some of the acutely ill patients from the CMHC region, and to some extent the differences in program profile may be functional. On the other hand, it may seem that the more family- and GP-oriented treatment is more functional for patients in need of long term follow up. This is in accordance with what is found in earlier studies [1].

**Figure 1:**
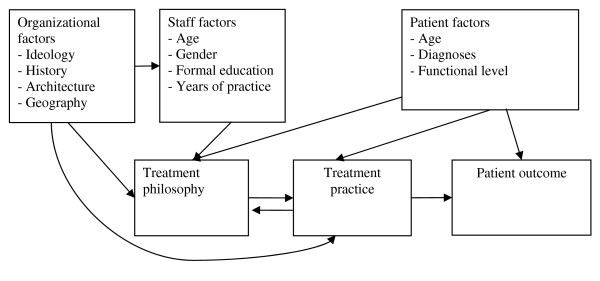
**Possible factors influencing outcome in mental health services**.

What determines treatment practice in the mental health field? [3]. Is it mainly socio-political trends, the structure or history of organizations, geography, the patient population or the staff's age, professional background and experience? Treatment programs are to a large extent adjusted to different clinical types of patients. This is supposed to be a central determinant. But our data indicates that differences in staff competence and education, and organizational characteristics may influence treatment philosophy and consequently practice. On the basis of our results, we have created a hypothesis of the relationship of these variables (figure 1). If results from the present study can be generalized, there seems to be a tendency that hospitals give what could be called a more medically oriented treatment over longer time with emphasis on evaluation and coping with aggression and less emphasis on cooperation with municipal health and social services. Lack of tradition and geographical closeness to patient's network may give newer organizations like CMHC with more flexible organizational frames and younger staff with educations and training that implies other treatment philosophies an advantage over presumably more slow-to-change large hospital organizations. When it comes to treatment of severely ill patients this hypothesis must be tested further.

A central aim for health authorities is to give the population equal access to health services regardless of population density and geographical distances [6]. Our results may indicate that an unintended consequence of differences in organization models is that even if the aim of equal access to services is reached, the services given may be different. But comprehensive information is required on the characteristics of the patients that receive inpatient services in the two regions before firm conclusions can be made.

It is also an aim to give a coordinated health service to patients with the most severe mental illness close to their local network. Our study may indicate that patients with long term rehabilitation needs receive treatment that is different in respect of inpatient specialized service from the two catchment areas.

### Strength and limitations

Our material is based on a relatively small number of ward staff from several different units. But the response rate is high, and some differences concerning competence and treatment profiles are very distinct. Doctors and psychologists are not included in our analyses. This is due to their somewhat different roles in relation to CMHC bed units and hospital units. In hospital units their job is fully connected to treatment of inpatients, while in the CMHC their job is partly connected to inpatients and partly to outpatients. It would not be possible to distinguish to what extent their ratings had their source in outpatient or inpatient experience. Preliminary analyses of data including doctors, psychologists and other treatment personnel indicate that including them had minimal impact on the results. It is not possible to draw conclusions about quality of treatment on the basis of our data on competence and treatment orientation and there are always differences between what people say they do and what they actually do [17]. However, this affects both the hospital and the CMHC staff to the same degree. Closer knowledge of groups of patients receiving different treatment in the units would strengthen our knowledge about aspects of the differences. Data from other parts of the VELO study will contribute to this.

CPPS is used in projects in USA and Norway. It seems to be a useful and economical instrument for comparing psychiatric services and for evaluating the services over time. Further research with the scale and analyses of its psychometric strength based on accessible Norwegian data is needed. To our knowledge this is the first international publication of a study using inpatient ward staffs CPPS ratings.

## Conclusion

• How psychiatric services are organised may influence the formulation of the treatment philosophy. In spite of considerable overlap between the CMHC and the hospital staff with regard to attitudes and priorities, there appeared a distinct but not radical difference between the units' treatment profiles.

• The hospital staff to a larger degree conceptualized their work in terms of long term treatment with emphasis on handling aggression, evaluation and medication, whereas their CMHC-inpatient colleagues emphasised family therapy and contact with GPs.

• Somewhat surprisingly, on treatment philosophy all units rated help with socio-economic rights, out of office orientation, teamwork, user participation and medication as most important. Psychotherapy was given a low priority, with other therapeutic modalities (e.g. group therapy, skill training) in between.

Our study shows that organizing community mental health specialist services with or without access to locally situated beds makes a difference that might influence the mental health services given to patients and their families, and the support given to the municipital health service in relation to patients with similar psychiatric disturbances.

## Competing interests

The authors declare that they have no competing interests.

## Authors' contributions

All authors participated in the design of the study. SB, KS, RO and LM wrote the manuscript. SB, KS and RO performed the statistical analysis. All authors read and approved the final manuscript.
